# Unusual ivory-white gallstones identified during indocyanine green fluorescence-guided laparoscopic cholecystectomy: a rare case report

**DOI:** 10.3389/fmed.2026.1844464

**Published:** 2026-05-21

**Authors:** Longfei Wu, Congyan Chen, Yuan Tian, Xin Wang

**Affiliations:** 1 Department of General Surgery, West China Hospital, West China Xiamen Hospital, Sichuan University, Xiamen, Fujian, China; 2 Division of Pancreatic Surgery, Department of General Surgery, West China Hospital of Sichuan University, Chengdu, China

**Keywords:** cholelithiasis, gallstones, indocyanine green fluorescence-guided, ivory-white gallstones, laparoscopic cholecystectomy

## Abstract

Ivory-white gallstones represent a rare morphological variant of cholelithiasis, differing from typical cholesterol stones, black pigment stones, or brown pigment stones. Their unusual appearance and poorly understood pathogenesis make them an infrequently reported finding in clinical practice. This study presents a case of ivory-white gallstones discovered during indocyanine green (ICG) fluorescence-guided laparoscopic cholecystectomy. A 70-years-old male presented with a 6-years history of intermittent right upper quadrant pain, clinically diagnosed as chronic symptomatic cholecystitis. Preoperative Computed Tomography (CT) revealed unusually hyperdense foci (500–1200 HU) within the gallbladder, suggesting high calcium content. The patient underwent indocyanine green (ICG) fluorescence-guided laparoscopic cholecystectomy. Gross examination revealed multiple distinctive ivory-white, brittle gallstones, with the largest measuring 2.5 cm. An intriguing “gel-to-solid” phase transition was observed: opalescent, mucoid-like concretions within the gallbladder cavity rapidly hardened into solid, chalky granules within minutes of exposure. Morphologically, the stones exhibited a homogeneous white interior without layering. While the absence of formal compositional analysis limits definitive mechanistic conclusions, the macroscopic appearance and high CT density suggest a complex interplay between cholesterol supersaturation and significant calcium deposition within a chronic inflammatory environment. This case highlights a rare morphological variant of cholelithiasis. Although such atypical morphology does not alter standard surgical management, it provides critical insights into the diverse physical chemistry of gallstone formation.

## Introduction

Gallstone disease is a common hepatobiliary disorder affecting approximately 10%–20% of the adult population worldwide (1). The formation of gallstones is a complex process involving supersaturation of bile components, nucleation of crystals, and impaired gallbladder motility (2). Factors including age, gender, weight, and bacterial infection also contribute to gallstone pathogenesis (3, 4). Based on their chemical composition, gallstones are generally classified into three main categories: cholesterol stones, black pigment stones, and brown pigment stones (5).

Despite the high clinical incidence of cholelithiasis, gallstones may rarely exhibit unusual phenotypic characteristics, presenting with distinctive colorations that diverge from established classifications. One such rare presentation is ivory-white gallstones (6). Ivory-white gallstones have seldom been reported in the literature. Herein, we report a rare case of ivory-white gallstones discovered during indocyanine green (ICG) fluorescence-guided laparoscopic cholecystectomy and discuss the possible mechanisms underlying this unusual presentation. This case report was organized and presented in accordance with the CARE international clinical (Supplementary material).

## Case presentation

A 70-years-old male presented to our hospital with a 6-years history of intermittent right upper quadrant abdominal pain. The episodes were predominantly postprandial, often triggered by high-fat meals, and typically lasted 2 h before resolving spontaneously. This clinical pattern was consistent with recurrent biliary colic. He denied fever, jaundice, nausea, or vomiting. Past medical history was significant for chronic hypertension and hyperlipidemia. The patient’s dietary habits were characterized by a prolonged and excessive intake of greasy and high-fat foods. Additionally, he had undergone a right knee arthroplasty 3 years prior. There was no history of hemolytic disease or previous hepatobiliary surgery. Physical examination revealed mild tenderness in the right upper quadrant without signs of peritonitis. Laboratory results, including liver enzymes and bilirubin, were within normal limits.

**FIGURE 1:**
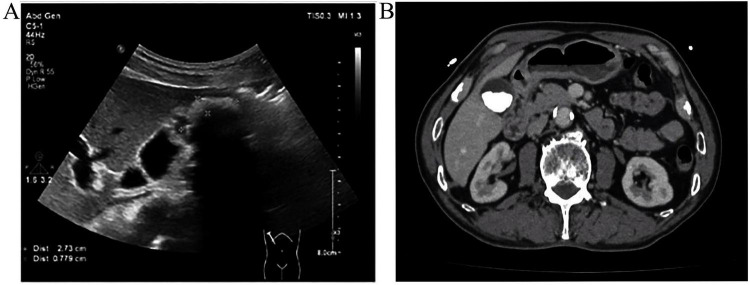
Preoperative imaging findings. **(A)** Ultrasonographic imaging showing hyperechoic gallstones with posterior acoustic shadowing; **(B)** Axial CT sections revealed the gallbladder lumen to be occupied by several fused, hyperdense foci (500–1200 HU), consistent with the subsequent macroscopic finding of ivory-white stones.

**FIGURE 2:**
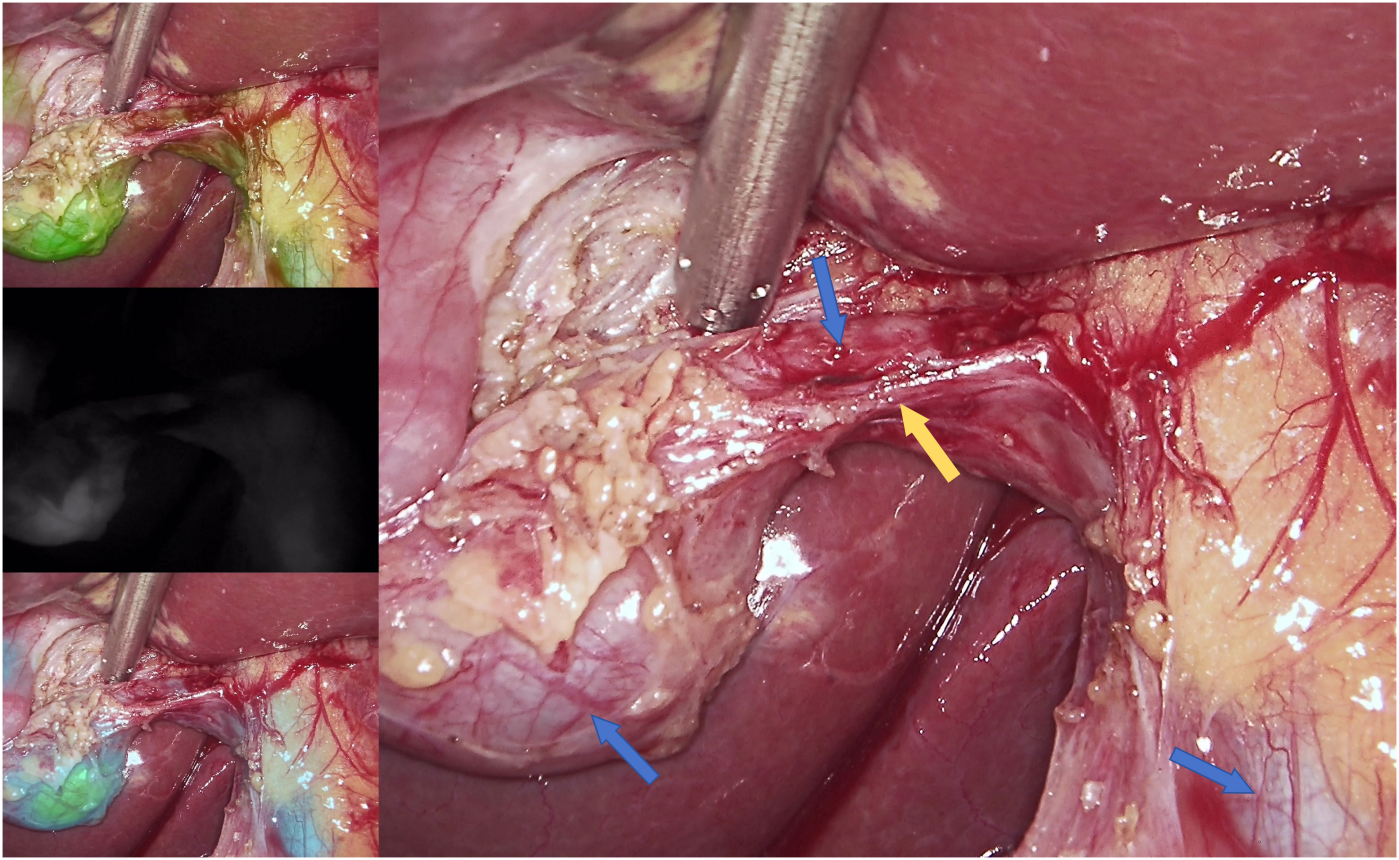
Indocyanine green (ICG) fluorescence-guided laparoscopic cholecystectomy. The cystic duct and common bile duct are clearly demarcated by ICG fluorescence. The blue arrows from left to right represent the gallbladder, the cystic duct, and the common bile duct, respectively; the yellow arrow represents the cystic artery.

Ultrasonography confirmed multiple echogenic structures with posterior shadowing and a mildly thickened gallbladder wall (Figure 1A). Computed Tomography (CT) plain scan revealed multiple conglomerated hyperdense foci within the gallbladder lumen. These lesions exhibited attenuation values ranging from 500 to 1200 HU (Figure 1B). Based on these findings, the patient was diagnosed with cholelithiasis. An ICG fluorescence-guided laparoscopic cholecystectomy was performed under general anesthesia using the three-port technique (Figure 2).

**FIGURE 3:**
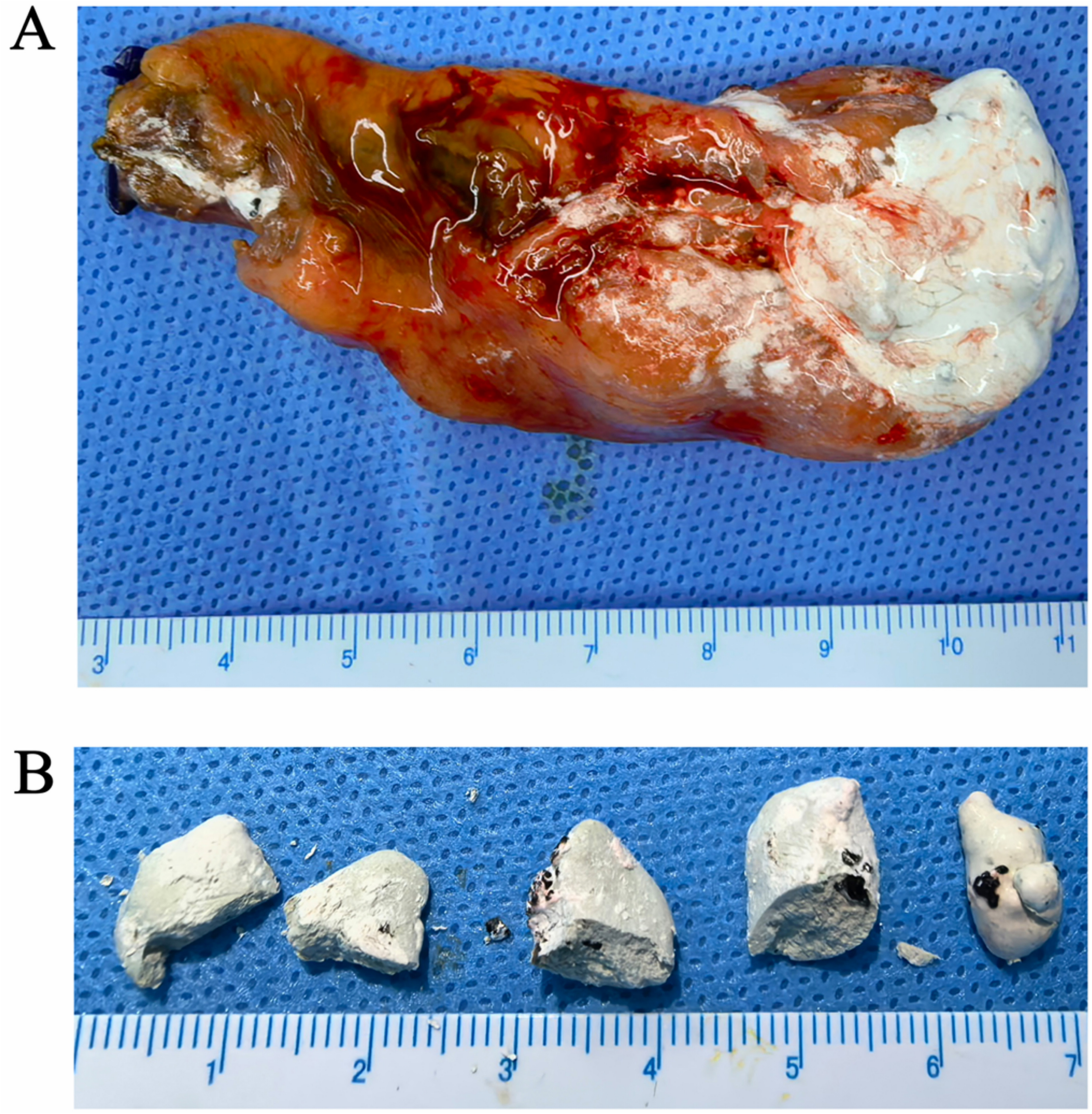
Gross appearance of the surgical gallbladder specimen. **(A)** Upon longitudinal incision, opalescent, mucoid-like concretions within the gallbladder cavity; **(B)** multiple distinctive ivory-white, brittle gallstones were identified within the lumen. The largest calculus measured 2.5 cm in diameter.

**FIGURE 4:**
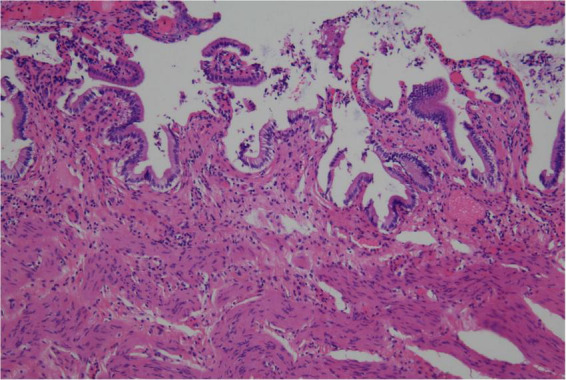
The postoperative pathological results.

Upon opening the excised gallbladder, we observed a mildly thickened wall consistent with chronic inflammation. The lumen contained several distinct ivory-white stones and viscous, opalescent mucoid-like material (Figure 3A). Notably, this mucoid material exhibited a “gel-to-solid” transition, rapidly hardening into brittle, chalky granules within minutes of atmospheric exposure. The retrieved stones exhibited rough surfaces and a brittle texture, characterized by a uniform ivory-white coloration. The largest specimen measured approximately 2.5 cm in diameter without the radial layering typical of pure cholesterol stones (Figure 3B). Gross examination revealed a homogeneous white cut surface. On sectioning, the stones demonstrated a homogeneous white interior without obvious layering or pigmentation. Histopathological examination of the gallbladder revealed features consistent with chronic cholecystitis, including mucosal atrophy, inflammatory cell infiltration. No evidence of dysplasia or malignancy was identified (Figure 4).

The patient recovered uneventfully and was discharged on postoperative day three.

## Discussion

Gallstones are classically categorized into cholesterol and pigment stones (7, 8). The “ivory-white” phenotype reported here represents a rare morphological variant, likely arising from extensive mineral sequestration within a predominant cholesterol matrix. Ultrasonography is the gold standard for cholelithiasis, offering >95% sensitivity. Typical findings include hyperechoic foci with distal acoustic shadowing and gravitational shift (9–12). Although CT superiorly depicts calcified gallstones, its overall sensitivity is limited as only 15%–20% of stones are calcified (13). The high CT attenuation values (500–1200 HU) observed in this case are significantly higher than those typical for pure cholesterol stones, which usually appear hypo-attenuating or isodense relative to bile. This discrepancy suggests that while the stone’s “ivory-white” exterior might be driven by a high cholesterol matrix, the core or internal structure is heavily impregnated with calcium salts (such as calcium carbonate or phosphate), as CT values exceeding 400 HU are highly predictive of significant calcification.

Similar cases of white gallstones have been sporadically documented. Araydah et al. (14) reported a case of a 44-years-old female diagnosed with cholelithiasis, where several unusual pearl-white calculi were identified during cholecystectomy. Subsequent quantitative analysis revealed that these stones consisted almost exclusively of cholesterol (99.6%), with negligible amounts of calcium carbonate (0.4%).

A defining feature of the present case was the intraoperative observation of a “gel-to-solid” transition. We propose that the gallbladder contents existed in a mucin-rich precursor phase or as advanced biliary sludge before rapidly hardening upon atmospheric exposure. Previous research demonstrated that gallbladder mucin acts as a “molecular glue” and a scaffolding matrix during stone nucleation, particularly within the chronic inflammatory microenvironment observed in our patient. It is plausible to hypothesize that the rapid transition reflects the physical dehydration or molecular cross-linking of this concentrated mucin scaffold when removed from the biliary environment (15, 16).

Furthermore, this transition aligns with the liquid-to-solid crystalline phase shifts described by Afdhal et al. (17). These observations potentially point to a “transitional state” of gallbladder contents–a snapshot of the active aggregation phase where lipid crystals are organized into solid calculi by a proteinaceous matrix. Although the lack of real-time chemical monitoring remains a limitation, these descriptive findings provide novel insights into the dynamic physical chemistry of atypical lithogenesis, moving beyond simple static morphological classification.

Infrared spectroscopy has enabled the classification of rare stone types, including those composed of calcium carbonate, calcium phosphate, and calcium fatty acids (18). Systematic research has shown that gallstone morphology and architecture vary by type: cholesterol stones feature a brownish-yellow hue and radial layering, whereas pigment and phosphate stones are black and amorphous (5, 19, 20). Ultrastructurally, scanning electron microscopy (SEM) reveals pathognomonic stacked plate-like crystals in cholesterol stones, contrasted with the bulbiform or acicular geometries of calcium carbonate stones. Elemental spectroscopy further confirms that specific mineral distributions, rather than ubiquitous carbon and oxygen, define each stone type (5, 21). Although formal chemical analysis was omitted, the extreme CT attenuation (1200 HU) provides a reliable surrogate indicator of significant calcification, which, when combined with the ivory-white gross morphology, supports a high-cholesterol/calcium-mixed composition. We hypothesize that the patient’s long-standing hyperlipidemia and high-fat diet created a state of cholesterol supersaturation, while the histopathologically confirmed chronic cholecystitis likely provided the inflammatory environment and pH shifts necessary for calcium precipitation. Although this single-case correlation remains speculative, the findings suggest that this rare phenotype may arise from a complex interplay between systemic metabolic dysregulation and localized gallbladder inflammation, rather than purely local biliary factors.

While morphologically distinctive, it does not appear to significantly alter the clinical presentation or management of gallstone disease. Imaging modalities including ultrasonography remain the primary diagnostic tools, although the color and internal composition of gallstones are generally not distinguishable on routine imaging. From a surgical perspective, however, the extreme hyperdensity identified preoperatively should alert the clinician to the high mineral content of the stones. Such physical properties might pose unique technical challenges, necessitating more meticulous handling during cystic duct to mitigate the risk of stone fragmentation or bile duct injury. Laparoscopic cholecystectomy remains the standard treatment for symptomatic gallstones and was successfully performed in this patient (22). The postoperative course was uneventful, and no additional interventions were required.

## Conclusion

In conclusion, ivory-white gallstones represent a rare morphological variant that likely arises from the interplay between high cholesterol saturation and chronic biliary inflammation. Although surgical intervention remains the gold standard, the striking nature of these stones provides an opportunity for clinicians to consider the patient’s broader metabolic profile. Presenting as a possible extreme manifestation of lipid dysregulation, this phenotype highlights the potential of using gallstone morphology to better understand and manage the systemic metabolic health of affected individuals.

## Data Availability

The original contributions presented in this study are included in this article/Supplementary material, further inquiries can be directed to the corresponding author.
